# Using Molecular Biology to Maximize Concurrent Training

**DOI:** 10.1007/s40279-014-0252-0

**Published:** 2014-10-30

**Authors:** Keith Baar

**Affiliations:** Functional Molecular Biology Lab, Department of Neurobiology, Physiology, and Behavior, University of California Davis, One Shields Ave, 174 Briggs Hall, Davis, CA 95616 USA

## Abstract

Very few sports use only endurance or strength. Outside of running long distances on a flat surface and power-lifting, practically all sports require some combination of endurance and strength. Endurance and strength can be developed simultaneously to some degree. However, the development of a high level of endurance seems to prohibit the development or maintenance of muscle mass and strength. This interaction between endurance and strength is called the concurrent training effect. This review specifically defines the concurrent training effect, discusses the potential molecular mechanisms underlying this effect, and proposes strategies to maximize strength and endurance in the high-level athlete.

## Introduction

Robert Hickson was a power-lifter when he went to do his post-doctoral work in the laboratory of Professor John Holloszy. Every day, Holloszy, the father of endurance exercise research, would leave the Washington University Medical Campus and go for runs through the adjoining Forest Park. In his effort to make a good impression with his new boss, Dr. Hickson decided to accompany Prof. Holloszy on his afternoon runs, but soon found that his muscle mass and strength were decreasing in spite of the fact that he was still doing his strength training at the same frequency and intensity. When Hickson approached Holloszy with his problem, he was told: “this should be the first study you do when you have your own lab.” True to his word, the first study that Hickson completed in his new laboratory at the University of Illinois in Chicago was the seminal study on concurrent training.

Published in 1980 [1], Hickson’s classic study trained three groups of subjects: Group 1 performed strength training alone; Group 2 performed endurance training alone; and Group 3 performed strength and endurance together. The strength training was performed 5 days per week for 10 weeks, and was designed exclusively to increase leg strength. True to his power-lifting background, Hickson had his subjects perform all of the exercises with as much weight as possible. The endurance training was performed 6 days per week for the same 10-week period and consisted of 3 days of cycling and 3 days of running. The cycling exercise consisted of six 5-min intervals at maximal aerobic capacity (VO_2max_), whereas the instructions on the running days were to “run as fast as possible” for 30 min/day in the first week, 35 min/day for the second week, and 40 min/day for the remainder of the study. The concurrent training group performed both the strength and endurance training protocols in a non-standardized order with between 15 min and 2 h of rest in between.

At the end of the 10-week training program, VO_2max_ was determined on the bike and treadmill. The strength alone group showed a 4 % improvement in VO_2max_ on the bike with no change when measured on the treadmill. In contrast, the endurance and concurrent training groups both increased VO_2max_ by 17 % on the treadmill and ~20 % on the bike. This indicated that strength training does not negatively affect endurance adaptations or performance. It should be noted, however, that the concurrent training group did not increase their bodyweight over the training period as a result of their strength training. If they had, it would be expected that their endurance performance could be affected, especially during running where they would have to support and propel this extra mass.

**Fig. 1:**
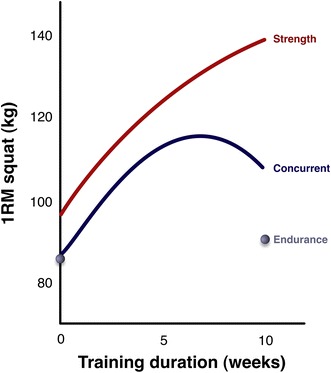
The concurrent training effect on strength. The figure shows the increase in one repetition maximum in the squat in subjects who participated in 10 weeks of high-intensity resistance exercise alone (resistance), endurance exercise alone (endurance), and both types of training (concurrent). Also, note that the strength and concurrent groups both increased their strength together up to 7 weeks, when the strength group started making greater gains than the concurrent group (adapted from Hickson [1], with permission). *1RM* one repetition maximum

As for strength, average strength in the concurrent and strength training groups increased at the same rate throughout the first 6–7 weeks of training (Fig. 1). Strength continued to increase throughout the entire 10-week training period in the strength training only group. In contrast, strength leveled off between the 7th and 8th weeks in the concurrent training group and surprisingly decreased during the 9th and 10th weeks of training. This indicates either that the concurrent training group was over-reaching or that high-intensity endurance exercise of a sufficient frequency can inhibit long-term strength adaptations.

When others have repeated the frequency and intensity that Hickson employed in his study, they have found a similar attenuation in strength and, importantly, impaired muscle fiber hypertrophy [2, 3]. For example, Kraemer and colleagues [2] showed that running and strength training at a high intensity for 4 days a week resulted in lower power concomitant with impaired muscle fiber hypertrophy than training for strength alone. Strength training alone resulted in ~28 % hypertrophy, whereas concurrent training resulted in only a ~16 % hypertrophy. This indicates that concurrent endurance training impairs not only strength but muscle hypertrophy as well.

It is important to note, though, that when the frequency, intensity, or duration of training is decreased, the degree of interference decreases. For example, in two separate studies McCarthy and colleagues showed that cycling 3 days a week for 50 min at 70 % VO_2max_ was not enough to impair strength [4] or hypertrophy [5] as a result of concurrent strength training. Further, Sillanpää et al. showed that cycling for 30 min twice a week below anaerobic threshold was not enough to impair strength or lean mass in middle-aged women [6] or old men [7]. Taken together, these data suggest that strength and endurance increase concomitantly up to a point. However, once the frequency increases past 4 days a week or the intensity of endurance exercise increases above 80 % VO_2max_, endurance exercise prevents the increase in muscle mass and strength that occurs with strength training. This was illustrated nicely in a recent meta-analysis that demonstrated that the effect size of strength training alone on muscle hypertrophy was 1.22 and for strength was 1.71 [3]. The corresponding numbers for concurrent training were 0.8 and 1.28, indicating that, in a large cohort, endurance exercise impairs muscle size and strength adaptations [3].

## Molecular Underpinning of Muscle Hypertrophy

Increased strength is the combined effect of improvements in neural activation, muscle fiber size, and connective tissue stiffness. Therefore, concomitant endurance exercise could decrease adaptations of any/all of these physiological parameters. There does not appear to be a decrease in the neural (learning) adaptation since in the early stages of training, when the neural adaptation is the strongest (4, 6, and 8 weeks), strength is similar between strength and concurrent training groups [1, 2]. However, it is possible that neuromuscular fatigue plays a role in the decrease in force as training continues. At this point, no one has measured the effect of concurrent training on connective tissue stiffness, so we are unsure of the role of this tissue in the impaired strength response. In contrast, as stated above, there is some evidence that muscle hypertrophy is impaired in individuals training for both strength and endurance together compared with those training exclusively with strength exercises, and that this correlates quite well with the impaired strength response [2, 3]. Therefore, the primary effect of endurance exercise seems to be a decrease in resistance exercise-induced muscle hypertrophy.

Over the last 15 years we have begun to understand the molecular events that lead to muscle hypertrophy and increased endurance capacity. These studies have shown that for exercise-induced muscle hypertrophy, the key signaling molecule is the mechanistic or mammalian target of rapamycin (mTOR). mTOR is a serine/threonine protein kinase that exists in two complexes. Both complexes contain the DEP domain-containing mTOR-interacting protein (DEPTOR) and the G-protein beta subunit-like protein (GβL; also known as lsT8). These proteins are negative and positive regulators of mTOR, respectively. Complex 1 (mTORC1) also contains the proline-rich Akt substrate of 40 kDa (PRAS40), an inhibitor of mTORC1 activity and the regulatory-associated protein of mTOR (raptor), which specifies the substrates that are phosphorylated by mTORC1. Raptor identifies the substrates for complex 1 by binding to TOS (TOR signaling) motifs, a five amino-acid sequence, found in proteins such as eukaryotic initiation factor (eIF) 4E binding protein-1 (4E-BP1), the 70-kDa ribosomal protein S6 kinase (S6K1), hypoxia-inducible factor-1 (HIF-1), and PRAS40. In contrast, complex 2 (mTORC2) contains the mammalian stress-activated map kinase-interacting protein 1 (mSIN1), which is important for targeting to membranes, the scaffold protein observed with rictor (PROTOR), and the rapamycin-insensitive companion of mTOR (rictor). Much like raptor in complex 1, rictor identifies the substrates that are phosphorylated by mTOR. However, rictor does not recognize TOS motifs and, as a result, in complex 2 mTOR is directed towards a completely different group of proteins including akt/PKB (protein kinase B), serum- and glucocorticoid-induced protein kinase (SGK), and protein kinase C (PKC). Importantly, the macrolide immunosuppressive antibiotic rapamycin specifically inhibits complex 1, allowing researchers to distinguish between the two complexes.

Following resistance exercise there is a significant and sustained increase in the activity of mTORC1, as determined by an increase in S6K phosphorylation [8] and activity [9]. The first indication that this increase in mTOR activity was important for resistance exercise-induced muscle hypertrophy came from work where we showed that the activity of mTOR 6 h following resistance exercise correlated with the increase in muscle mass following 6 weeks of training [8]. This finding in rats has since been demonstrated in humans [10], suggesting that activation of mTORC1 is key to increasing muscle mass and strength.

**Fig. 2:**
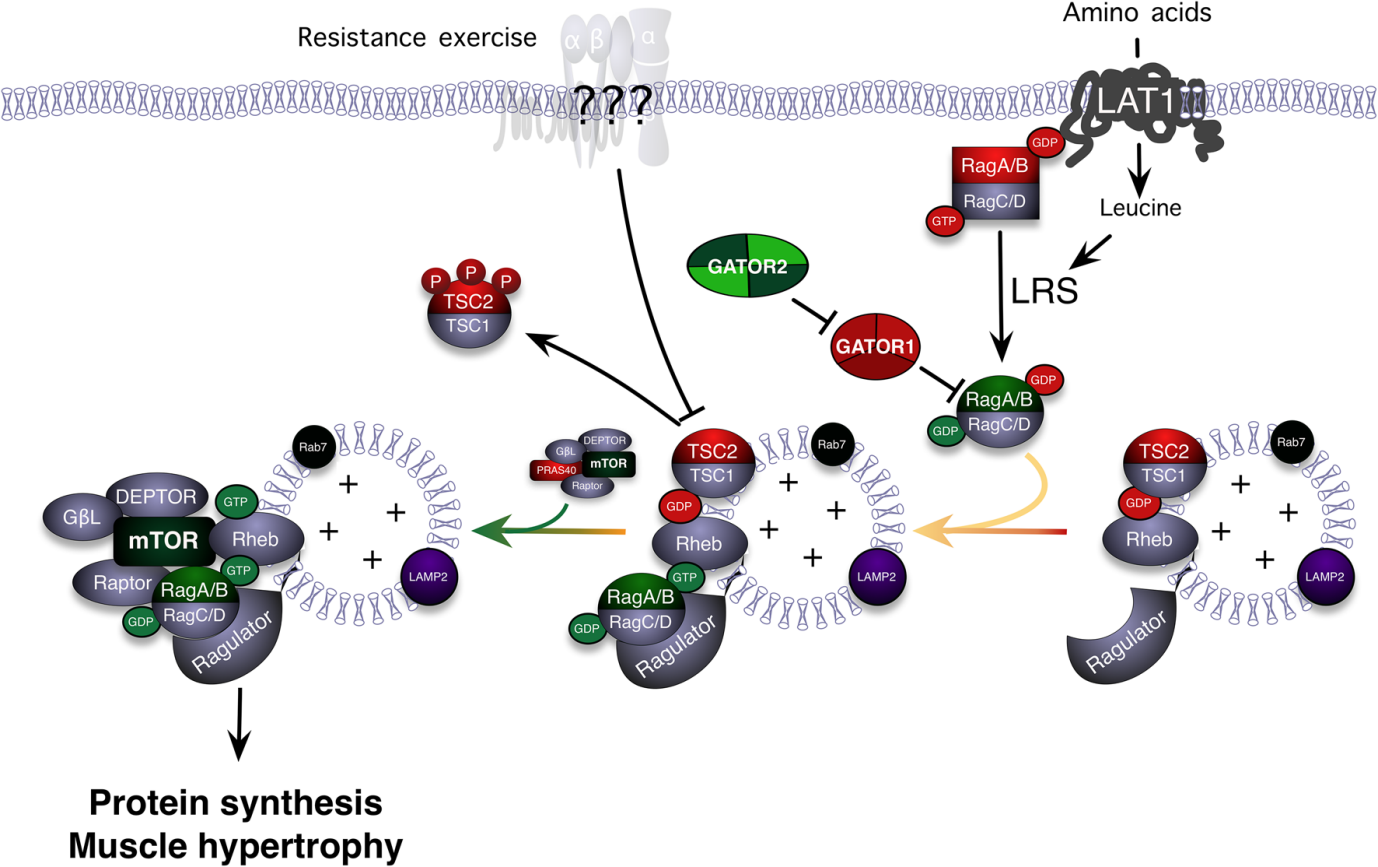
The activation of the mechanistic target of rapamycin complex 1 (mTORC1, mTOR, and raptor complex) following resistance exercise and feeding. Lifting a heavy weight to failure stimulates a mechanoreceptor that in turn activates an RxRxxS*/T* kinase (depicted by *???* at the membrane) that phosphorylates and moves the TSC2 away from the lysosome allowing Rheb to remain in the GTP bound state. Simultaneously, amino acid uptake and intracellular amino acid levels increase. The extra amino acids stimulate the LRS to act as a GAP towards RagC/D and GATOR2 blocks GATOR1 (the GAP of RagA/B) and the Ragulator GTP loads RagA/B and activates the complex. The active Rag complex then binds to raptor and positions mTOR beside its activator: GTP bound Rheb. The resulting elevation of mTORC1 activity drives myofibrillar protein synthesis and eventually leads to an increase in muscle mass and strength. *DEPTOR* DEP (Dishevelled, Egl-10 and Pleckstrin) domain-containing mTOR-interacting protein, *GβL* G-protein beta subunit-like protein, *GAP* GTPase activating protein, *GATOR* GAP Activity Towards Rags, *GDP* guanosine diphosphate, *GTP* guanosine triphosphate, *LAMP2* lysosome-associated membrane protein 2, *LAT1* L-type amino acid transporter, *LRS* leucyl transfer RNA synthase, *mTOR* mammalian target of rapamycin, *mTORC1* mTOR complex 1 *P* phosphorylation, *PRAS40* proline-rich Akt substrate of 40 kDa, *Rab7* Ras-related protein 7, *raptor* the regulatory-associated protein of mTOR, *Rheb* Ras homolog enriched in brain, *TSC2* tublerosclerosis complex

In many cells, mTOR is activated by growth factors as a way to stimulate protein synthesis [11]. However, resistance exercise activates mTOR in a growth factor-independent manner [12]. Unlike growth factors that use a receptor tyrosine kinase to signal through phosphoinositide 3-kinase (PI3K) to PKB, resistance exercise activates mTOR without activating PI3K [13]. Instead, resistance exercise activates an unidentified kinase (Fig. 2) that phosphorylates the potent mTOR inhibitor tuberin (TSC2) on RxRxx motifs [14]. When TSC2 is phosphorylated in this manner, it binds to 14-3-3 proteins and is moved away from mTOR and its activator Ras-homolog enriched in brain (Rheb). In this way, Rheb becomes activated and stimulates mTORC1 activity, leading to increased protein synthesis.

Contraction-induced dissociation of TSC2 from the lysosome is not the only thing that occurs following resistance exercise that leads to the prolonged activation of mTOR. In the hours after resistance exercise there is also an increase in the rate of amino acid uptake from the blood into the muscle. Specifically, leucine and glutamine are increased within the working muscle [9, 15]. This increase in leucine within the muscle is likely the result of an increase in the primary leucine transporter (LAT1) messenger RNA (mRNA) [16] and protein [17]. Interestingly, the increase in glutamine can help drive leucine uptake since LAT1 transports glutamine out of the muscle as it transports leucine into the muscle in a process called tertiary active transport [18]. As leucine enters the muscle it acts to trigger protein synthesis largely through its ability to activate mTORC1 [19]. As leucine is taken up, it binds to the leucyl-transfer RNA (tRNA) synthetase (LRS). This not only conjugates leucine to its tRNAs, but the LRS also acts as the first step in the amino acid activation of the mTORC1 [20]. LRS may serve as a GTPase activating protein (GAP) towards the small G-protein (RagD), which in turn is a component of a heterodimer of RagA/B and RagC/D that is important for amino acid sensing. When RagD is bound to guanosine triphosphate (GTP) it forms an inactive complex. LRS catalyzes the hydrolysis of this GTP to guanosine diphosphate (GDP) and RagD then interacts with the Ragulator [21]. At the Ragulator, the Rag heterodimer interacts with GATOR (GAP Activity Towards Rags), an octomeric complex that controls the activity of the Rag proteins [22]. In the presence of amino acids, the Rags are activated and recruit mTORC1 to the lysosome by binding to raptor [23, 24]. As discussed above, the activator of mTOR, Rheb, is also located on the lysosome, so the net effect of leucine is to bring mTOR to its activator.

Together, these data suggest that resistance exercise activates mTORC1 through the activation of an RxRxx-directed kinase that phosphorylates and moves the inhibitor TSC2 away from the lysosome (Fig. 2). At the same time, by regulating LAT1, enhanced amino acid uptake moves mTOR to the lysosome where it can be activated by GTP-bound Rheb. This complex molecular process explains both the load-dependent activation of mTOR (more activation of the RxRxx kinase [14]) and the effects of amino acid consumption (increased movement of mTOR to the lysosome and its activator Rheb [23, 24]) on protein synthesis and, finally, muscle hypertrophy [25, 26].

Even though a molecular mechanism exists for the activation of mTORC1 by resistance exercise and protein feeding, this does not prove that mTORC1 is what is necessary for muscle growth. Evidence that mTORC1 drives load-induced muscle hypertrophy was derived from experiments using rapamycin to specifically inhibit the kinase. In mice, Bodine and colleagues showed that daily injections of rapamycin could inhibit muscle hypertrophy [27], and in humans, Drummond and colleagues showed that rapamycin could block the acute increase in protein synthesis after resistance exercise [28]. These experiments suggested that a rapamycin-sensitive process was required for load-induced muscle growth. The specific role of mTOR was demonstrated by an elegant study from the Hornberger laboratory using mice with a knock-in mutation that made mTOR resistant to treatment with rapamycin [29]. As would be expected, in the wild-type animals rapamycin completely blocked muscle hypertrophy. However, in the mice expressing a rapamycin-resistant mutant of mTOR muscle, hypertrophy occurred normally both in the presence and absence of rapamycin [29]. These data showed conclusively that resistance exercise-induced muscle hypertrophy is completely dependent on mTOR. Therefore, research looking for a molecular mechanism underlying the concurrent training effect has been focused exclusively on ways that endurance exercise could inhibit mTORC1 activity.

## Molecular Underpinning of Enhanced Endurance

Whereas the muscle hypertrophy response to resistance exercise appears to converge on a single protein complex (mTOR), endurance adaptations are the result a variety of metabolic signals and molecules. During endurance exercise the concentration of calcium, oxygen free radicals, adenosine monophosphate (AMP), lactate, nicotinamide adenine dinucleotide (NAD^+^), inorganic phosphate, and glycogen change dramatically within the working muscle. At the same time, systemic changes in hormones, such as catecholamines, may influence the muscle and drive adaptations. These metabolic and hormonal signals turn on signaling proteins within muscle that, if repeated at a sufficient frequency, lead to enhanced mitochondrial mass, improved fat and glucose oxidation, and increased capillary density. For example, calcium released during contraction activates the calcium-calmodulin kinase (CaMK) family of proteins, specifically CaMKII in skeletal muscle [30]. Active CaMK can increase both the capacity for glucose uptake through upregulation of the glucose transporter GLUT4 [31], and mitochondrial mass by transcriptional upregulation of the mitochondrial biogenesis regulator PGC-1α (peroxisome proliferator γ coactivator 1α) [32]. The decrease in adenosine triphosphate (ATP) and glycogen and the rise in adenosine diphosphate (ADP) and AMP during high-intensity endurance exercise activates the AMP-activated protein kinase (AMPK). Active AMPK is involved in the increase in fat oxidation during exercise [33] and also plays a role in the long-term regulation of mitochondrial mass by controlling the transcription and activity of PGC-1α [34]. The decrease in glycogen also activates the 38 kDa mitogen-activated protein kinase (p38), which, like AMPK, can increase the transcription and activity of PGC-1α [35–37]. The rise in lactate and NAD^+^ activates the NAD^+^-dependent deacetylase family of sirtuins (SIRT). Members of this family control metabolic flux through the tricarboxylic acid (TCA) cycle, insulin sensitivity [38], and PGC-1α activity [39]. Last, the rise in circulating catecholamines through the β-adrenergic receptor activates the cyclic AMP response element binding protein (CREB), a transcription factor that is required for the transcriptional upregulation of PGC-1α [40].

Since all of these signaling molecules are activated by endurance exercise, it is possible that one or more of them can simultaneously inhibit mTOR activation and limit skeletal muscle hypertrophy during concurrent training. Beyond these signals, which are known to play a positive role in the adaptation to endurance exercise, the stress of exercise is known to increase other processes, such as free radical generation or endoplasmic reticulum (ER) stress [41, 42] that might influence mTOR activity or protein synthesis in response to resistance exercise.

## Current Data on the Molecular Underpinning of the Concurrent Training Effect

The first hint of a molecular mechanism that could explain how endurance exercise could impair muscle hypertrophy of concurrent strength training came when Inoki and colleagues showed that metabolic stress blocks mTORC1 activity [43]. Of keen interest for exercise physiologists was the fact that AMPK was required for the inhibitory effect of metabolic stress on mTOR [43]. The effect of metabolic stress on mTOR was first suggested to be the result of AMPK phosphorylating and activating the mTOR inhibitor TSC2 [43]. Later, another group showed that AMPK could phosphorylate raptor and dissociate the mTORC1 [44]. More recently, a third group has shown that in some cells, metabolic stress inhibits mTOR in an AMPK-independent manner by preventing mTOR localization to the lysosome [45].

Regardless of the mechanism, putting together the effect of metabolic stress/AMPK activation on mTOR and the fact that metabolic stress and AMPK activity were increased during endurance exercise, exercise physiologists began to ask the question “can AMPK limit muscle hypertrophy?” Thomson and Gordon were the first to show that impaired muscle growth was seen in rats where AMPK activity was higher, supporting the hypothesis that AMPK mediated the concurrent training effect [46]. They went further using the AMP mimetic AICAR (5-aminoimidazole-4-carboxamide ribonucleotide) to activate AMPK in muscles before resistance exercise and, consistent with the hypothesis, AICAR treatment blocked S6K phosphorylation [47]. We then used an animal model of concurrent training to show that the isoform of AMPK that was activated by concurrent training (α1) was not the form that was associated with endurance adaptations (α2), suggesting that during concurrent training a different form of AMPK was activated to prevent growth [48]. Consistent with the hypothesis that α1-AMPK limited growth, mice in which the α1 isoform of AMPK was knocked out showed a 33 % greater increase in muscle fiber size and enhanced mTOR signaling to S6K and 4E-BP1 in response to training than wild-type mice [49]. It is important to note that the α1-AMPK knockout mice showed significantly greater α2-AMPK activity in an effort to compensate for the loss of α1, but this was unable to restrict growth. Therefore, the metabolically activated α2 form of AMPK was not as effective at inhibiting mTOR as the α1 form.

Even though the animal studies have been impressive at showing that AMPK can directly inhibit mTORC1 activity and muscle growth, acute studies in humans are not as definitive. The most interesting of these studies is a pair from John Hawley’s laboratory [50, 51]. In the first, they showed that the activation of the mTORC1 following eight sets of five repetitions at 80 % of their one repetition maximum (1RM) was completely lost if the subjects had performed ten 6-second maximal sprint efforts on a bicycle 15 min before strength training, and mTOR activity rapidly returned to baseline if the sprint session was performed after strength training [50]. Interestingly, if, instead of using a high-intensity sprint session, the authors used a moderate intensity bout of cycling, there was no difference in mTORC1 activity [51]. Consistent with endurance exercise intensity being a key to the interference effect, Lundberg et al. did not find any inhibition of mTOR activation when subjects performed 45 min of cycling at 70 % VO_2max_ 6 h before performing resistance exercise [52]. Further, Apró and colleagues did not report any decrease in mTOR signaling when subjects performed 30 min of cycling at 70 % of VO_2max_ 15 min after completing a resistance training session [53]. These findings are completely consistent with the training data that show that the interference effect is only seen if the subjects train at a high frequency and intensity [1, 2], and the fact that the muscle AMP/ATP ratio and AMPK activity increases with exercise intensity [54]. Even though the intensity effects and the animal data are completely consistent with AMPK mediating the inhibition of mTOR activity during concurrent training, the activation of AMPK in both of the training groups was the same in the sprint interval study by Coffey and colleagues, suggesting that AMPK could not explain the inhibition of mTOR activity [50]. With the caveat that the phosphorylation of AMPK is not the most sensitive measure of AMPK activity (a direct measure of activity or the phosphorylation of its downstream target acetyl-CoA carboxylase show both the allosteric activation by AMP or ADP and the effect of phosphorylation [54]), this suggests that another molecular signal contributes to, or better explains, the inhibitory effect of endurance exercise on muscle hypertrophy.

## Alternative Molecular Underpinning of the Concurrent Training Effect

If the activation of AMPK does not completely explain the concurrent training effect, then what other molecular events are activated by endurance exercise that could block mTOR and/or inhibit muscle hypertrophy? As discussed in Sect. 3, endurance exercise activates the sirtuin family of NAD^+^-dependent deactetylases including SIRT1 [55]. Like AMPK, SIRT1 is activated by metabolic stress and as a result of its relationship with lactate/NAD^+^, is activated in an intensity-dependent manner [56]. Further, SIRT1 is able to inhibit mTOR [57]. In HeLa cells, where AMPK activity is reduced, the knockdown or inhibition of SIRT1 increased mTORC1 activity, whereas the SIRT1 activator resveratrol decreased mTOR activity [57]. Further, since SIRT1 and AMPK signaling are closely linked [58], it is possible that SIRT1 and not AMPK is the direct mediator of mTOR inactivation that was discussed above following high-intensity exercise.

Another way that endurance exercise could inhibit mTORC1 activity is through the unfolded protein, or ER stress, pathway. Periods of high lipid exposure, glucose deprivation, or increased synthesis of secretory proteins, lead to the accumulation of unfolded or misfolded proteins within the ER lumen [59]. To cope with the increase in unfolded proteins, cells activate the unfolded protein response, a series of events that serve to block general protein synthesis, increase protein-folding capacity, and restore ER function. Interestingly, the unfolded protein response is activated in muscle by acute endurance exercise [42], a high-fat diet [60], or the combination of both stimuli [41]. Furthermore, both endurance exercise [2] and a high-fat diet [61] impair muscle hypertrophy and ER stress decreases mTORC1 activity and protein synthesis in muscle [60, 62]. Therefore, like AMPK and SIRT1, the ER stress response could contribute to the concurrent training effect.

## Science-Based Recommendations for Training to Maximize Concurrent Training

Using the molecular information provided in Sects. 2–5, some simple nutritional and training strategies can be devised to maximize the adaptations to concurrent training. The goal of these recommendations is to maximize the mitochondrial adaptation to endurance exercise and the muscle mass and strength adaptation to strength training. To do this, the following could be recommended:

- Any high-intensity endurance training sessions should be performed early in the day. Then, a period of recovery of at least 3 h should be given, so that AMPK and SIRT1 activity can return to baseline levels, before resistance exercise is performed. This suggestion is based on the fact that AMPK activity increases rapidly and then returns to baseline levels within the first 3 h after high-intensity exercise [63], whereas mTORC1 activity can be maintained for at least 18 h after resistance exercise [8, 9].
- Resistance exercise should be supported by readily digestible, leucine-rich protein as soon as possible after training to maximize leucine uptake [64], mTOR recruitment to the lysosome [29], and protein synthesis [25]. Since, in this scenario, resistance exercise is performed later in the day, it becomes even more important to also consume protein immediately prior to sleep to maximize the synthetic response overnight [65].
- Fully refuel between the morning high-intensity endurance training session and the afternoon strength session since AMPK can be activated by low glycogen [66], and SIRT1 is activated by caloric restriction [38]. If it is not possible to refuel completely because of the training volume and intensity, it might be best to reserve a portion of the offseason (and short periods in season) exclusively for increasing muscle size and strength and then use higher dietary protein intakes to maintain that muscle mass as the aerobic load increases through the season [67].
- To improve the endurance response to lower-intensity endurance training sessions and provide a strong strength stimulus, consider performing strength training immediately after low-intensity, non-depleting, endurance sessions. Performing a strength session immediately after a low-intensity endurance session results in a greater stimulus for endurance adaptation than the low-intensity endurance session alone [68] and the low-intensity session will not affect signaling pathways regulating strength gains [51–53].

## Conclusions

These simple recommendations, based on our current understanding of the molecular response to exercise, should allow for the maximal adaptive response to both endurance and strength exercise. However, it is important to remember that what makes a good molecular biologist is the ability to break down complex physiological processes into simple molecular switches. Naturally, improving endurance and strength together in an elite athlete is more than just striking the balance between AMPK/SIRT1 and mTORC1. This is especially true in situations where performance is based on skill optimization that goes well beyond these simple molecular pathways. In the end, how an athlete performs with their improved endurance and strength is based on far more complex processes that are unfortunately poorly understood.
